# Suppression of self-absorption in laser-induced breakdown spectroscopy using a double pulse orthogonal configuration to create vacuum-like conditions in atmospheric air pressure

**DOI:** 10.1038/s41598-020-70151-6

**Published:** 2020-08-06

**Authors:** Indra Karnadi, Marincan Pardede, Ivan Tanra, Rinda Hedwig, Alion Mangasi Marpaung, Zener Sukra Lie, Eric Jobiliong, Dennis Kwaria, Maria Margaretha Suliyanti, Muliadi Ramli, Kurnia Lahna, Tjung Jie Lie, Hery Suyanto, Koo Hendrik Kurniawan, Kiichiro Kagawa

**Affiliations:** 1 Department of Electrical Engineering, Krida Wacana Christian University, Jakarta, 11470 Indonesia; 2 Department of Electrical Engineering, University of Pelita Harapan, Tangerang, 15811 Indonesia; 3 Computer Engineering Department, Faculty of Engineering, Bina Nusantara University, Jakarta, 11480 Indonesia; 4 Faculty of Mathematics and Natural Sciences, Jakarta State University, Jakarta, 13220 Indonesia; 5 Automotive and Robotics Program, Computer Engineering Department, Binus ASO School of Engineering, Bina Nusantara University, Jakarta, 11480 Indonesia; 6 Research Center of Maju Makmur Mandiri Foundation, Jakarta, 11630 Indonesia; 7 Research Center for Physics, Indonesia Institute of Science, Kompleks Puspiptek, Tangerang Selatan, 15314 Indonesia; 8 Chemistry Department, Faculty of Mathematics and Natural Sciences, Syiah Kuala University, Darussalam, Banda Aceh, 23111 Indonesia; 9 Physics Department, Faculty of Mathematics and Natural Sciences, Syiah Kuala University, Darussalam, Banda Aceh, 23111 Indonesia; 10 Department of Physics, Faculty of Mathematics and Natural Sciences, Udayana University, Kampus Bukit Jimbaran, Denpasar, 80361 Indonesia; 11 Fukui Science Education Academy, Takagi Chuo 2 Chome, Fukui, 910-0804 Japan

**Keywords:** Engineering, Optics and photonics, Physics

## Abstract

Self-absorption, which is known to severely disturb identification of the emission peak intensity in emission-based spectroscopy, was first studied using ordinary single pulse laser-induced breakdown spectroscopy (LIBS). It was found that severe self-absorption, with an evident self-reversal, occurs in the resonance emission lines of high concentration Na, K, and Al, and thus it is impossible to obtain the linear calibration curve required for quantitative analysis. To overcome this problem, we introduce a double pulse orthogonal technique in which the first laser is fired in a parallel orientation at a varied distance of 2–6 mm from the sample surface. It is well known that the strong shock wave generated by this laser irradiation temporarily creates a vacuum-like condition immediately in front of the sample surface. This action is followed by a second laser irradiation oriented perpendicular to the sample surface. The sample ablated by the second laser irradiation expands following the shockwave excitation process in the vacuum-like air atmosphere created by the first laser. The obtained spectra of the resonance emission lines of high concentration Na, K, and Al are free from the self-reversal and weakly affected by the self-absorption effect. A linear calibration curve that intercepts near zero point for K element over a wide concentration range is also demonstrated in this study. This simple modification is considered notably helpful in overcoming the self-absorption that occurs in ordinary single pulse atmospheric pressure LIBS.

## Introduction

The invention of pulsed laser has ushered a new research era on light-matter interaction ranging from laser material processing^1,2^ to laser-induced plasma spectroscopy. These days, laser-induced plasma spectroscopy for spectrochemical analysis has become a highly popular and sophisticated analytical tool since the first discovery was demonstrated by Brech and Cross^3^ in 1962. Researchers are drawn to the simplicity of this technique in generating laser-induced plasma, including its rapid and in situ analysis that requires little to no sample preparation. This technique also allows practical application of local micro-analysis and has the ability to capture real-time on-line analytical results^4,5^. Further developments of this technique follow two main directions. One direction adopts a high pressure surrounding gas and is referred as laser-induced breakdown spectroscopy (LIBS), which was first reported by Radziemski et al.^6,7^ The other direction adopts a low pressure surrounding gas to suppress the background emission intensity and self-absorption effect and is referred as laser-induced shockwave plasma spectroscopy (LISPS), which was first reported by Kagawa et al.^8,9^ However, in later development, this low pressure technique was not widely used due to its inapplicability to in situ experiments. Therefore, only the terminology of LIBS is currently used, even though the experiments are conducted in low pressure.

However, it should be noted that the well-known LIBS technique suffers from the effect of self-absorption, especially for emission lines originating from direct resonant transition involving the ground state and for high concentrations of the analyte atoms^4^. Therefore, at this condition, LIBS is inapplicable to quantitative analysis because it is not possible to obtain a linear calibration curve. Recently, many experiments have been conducted to suppress the self-absorption effect. For example, in their experiments, Ran Hai et al.^10^ found that the use of an argon atmosphere and proper selection of the detection window can effectively reduce the self-absorption effect. Zhang Xiong et al.^11^ used fibre laser LIBS to suppress self-absorption of the Mg and Ca emission lines. Yun Tang et al.^12^ improved the linearity of the Mn calibration curve in steel by choosing a proper detection window and also proposed microwave-assisted excitation in LIBS (MAE-LIBS) to reduce the effect of self-absorption over a wide spectral range of 200–900 nm^13^. Jia Ming et al.^14^ used laser-stimulated absorption LIBS (LSA-LIBS) to reduce self-absorption of the K, Mn, and Al emission lines. In addition to these experimental approaches, certain researchers used mathematical models to minimize the self-absorption effect^15–17^. Because of the importance of the self-absorption effect relative to the linearity of the calibration line in LIBS, many studies have also been proposed and cannot all be listed in this manuscript, but they can be considered as excellent references for the study of self-absorption^18–25^.

In our previous publications, which mainly used a single laser in a low pressure surrounding gas, we confirmed that no self-absorption is observed in the emission lines^26–40^. We also reported in another publication that the self-absorption effect can be significantly suppressed by applying a double pulse orthogonal configuration in atmospheric He gas. In this case, the excitation of the ablated target atoms is primarily due to the Penning-like energy transfer process between the He metastable excited-state atoms and the ablated target atoms^41^. Although the results are excellent, i.e., a linear calibration curve and narrow full width at half maximum (FWHM), the use of He gas is highly complicated and costly. Encouraged by the above results, we proposed a new double pulse orthogonal configuration in which the He gas is replaced by atmospheric air. In this case, the first laser is used to create a vacuum-like condition immediately in front of the sample surface, and the second laser is used to ablate the sample. Therefore, the ablated atoms from the target experience a vacuum-like condition during propagation, which is the same as in the case of low pressure plasma to a certain extent. By choosing the proper distance between the first laser irradiation spot and the sample surface and by adjusting the inter-pulse delay times between the two laser operations, we can obtain emission lines of high concentration K, Na, and Al that are practically free from the self-reversal and weakly affected by the self-absorption effect. A calibration curve for K element over a wide concentration range yielding an almost linear line that intercepts near zero point. It should be noted that this new double pulse technique is slightly different from the ordinary and well known double pulse technique. Specifically, in the pre-ablation double pulse technique, the observed intensity enhancement effect was commonly attributed to the rarefied/vacuum-like gas condition created by the pre-ablation laser pulse, which led to a more effective ablation process^42–44^. Thus the intent of the ordinary double pulse technique is to obtain a higher emission intensity, which is different with our double pulse technique.

## Materials and methods

The samples used in this experiment are KCl and NaCl powder (Wako Chemical, Japan, 4N) and standard Al samples containing 99.8% Al. The Cu sample from Rare Metallic Co. Japan is also used in this experiment. The powder samples were ground until the grain size was approximately 50 µm and subsequently pressed into pellets under a pressure of 30 MPa for approximately 90 s. The resulting pellet had a diameter of 10 mm and a thickness of 2 mm.

The experimental arrangement used in this work is provided in the *Supporting Information*. Figure S1(a), Supporting Information, shows the schematic diagram of the experimental setup, and Fig. S1(b), Supporting Information, shows the real setup of the apparatus. The system generally consists of a two-laser system as the irradiation source, a x–y–z manual stage sample holder and a high-resolution spectrograph for analysing the plasma emission from the target. The two-laser system is exactly the same as the one reported previously^41^, but it is described again in Fig. S1 for easy reference. The first laser is a Nd:YAG laser (Quanta Ray, LAB 130-10, USA, 10 Hz repetition rate) operating at a wavelength of 1,064 nm and a fixed energy of 122 mJ during the entire experiment. This laser is focused from above the sample holder with a quartz lens with 100 mm focal length to generate the air breakdown plasma approximately 6 mm from the target surface. In this stage, we confirmed the production of strong air breakdown by the occurrence of a strong white colour associated with the continuum emission, as shown in the inset of Fig. S1(a), Supporting Information. The second Nd:YAG laser (Quanta Ray, INDI 10, USA) operating at 355 nm with a 10 Hz repetition rate and energy of 20 mJ/pulse is used to ablate the target during the entire experiment. This laser is horizontally focused on the target surface using a quartz lens with 150 mm focal length to create the target plasma, as also shown in the inset of Fig. S1(a), Supporting Information. The emission spectra around the target plasma are collected by an optical fibre with one of its ends positioned 6 cm along the side from the air breakdown such that the fibre with a numerical aperture of 27° is able to collect the entire light emitted from the air breakdown and target plasma region, as shown in the inset Fig. S1(a), Supporting Information. The other end of the fibre is connected to the input slit of a spectrograph (Andor model 2061, focal length 1,000 mm, f/8.6, Czerny Turner configuration) with its exit slit coupled to a gated intensified charge-coupled device (ICCD; Andor iStar intensified CCD, 1,024 × 256 pixels, UK). The triggering of the ICCD and the synchronization between the two lasers are facilitated using a digital delay generator (DDG 535, Stanford Research System, USA). The gate delay and gate width of the ICCD are fixed at 200 ns and 30 µs, respectively, after the initiation of the second laser during all of the experiments. All experiments were conducted under the fixed sample position with 5 data accumulations.

## Results and discussion

**Figure 1:**
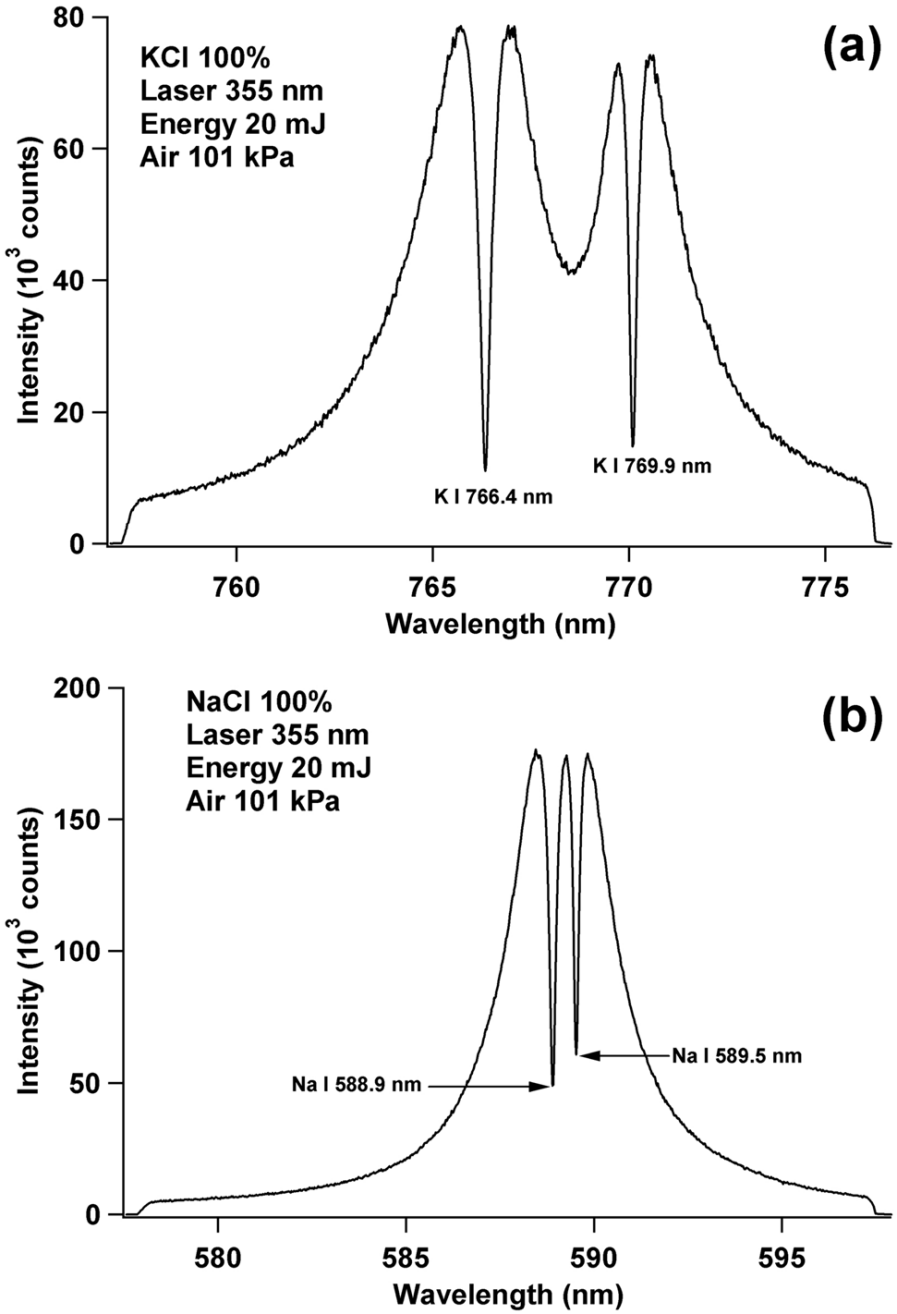
(**a**) Emission spectra of K I 766.4 nm and K I 769.9 nm from a pure KCl pellet sample and (**b**) emission spectra of Na I 588.9 nm and Na I 589.5 nm from a pure NaCl pellet sample when irradiated with a Nd:YAG laser at 355 nm wavelength and energy of 20 mJ. The ambient air pressure is 101 kPa. The gate delay and gate width of the ICCD are set at 200 ns and 30 µs, respectively.

Prior to the use of the double pulse orthogonal configuration, it is important to examine how the emission spectrum looks when the ordinary LIBS configuration is applied, i.e., using only a single pulse laser. For this purpose, we prepared two pellet samples of high-purity KCl and NaCl powders. Figure 1a shows the emission spectrum of the KCl pellet when irradiated with the 355 nm Nd:YAG laser operating at an energy of 20 mJ. The gate delay and gate width of the ICCD are fixed at 200 ns and 30 µs, respectively, as explained in the procedure. From Fig. 1a, severe self-absorption, with an evident self-reversal, is observed for the emission lines of K I 766.4 nm and K I 769.9 nm. The same case is also observed for the NaCl sample, i.e., severe self-absorption, with an evident self-reversal, is observed for the emission lines of Na I 588.9 nm and Na I 589.5 nm (Fig. 1b). Both experiments are conducted in atmospheric air pressure of 101 kPa. When the air pressure is reduced to 0.67 kPa, strong and narrow FWHM emission lines of K I 766.4 nm and K I 769.9 nm, with negligible self-absorption, are observed, as shown in Fig. S2(a), Supporting Information, and the same are also shown for the emission line of Na I 588.9 nm and Na I 589.5 nm in Fig. S2(b), Supporting Information. It should be noted that this component of the experiment is conducted using a vacuum chamber to evacuate and maintain the same air pressure of 0.67 kPa. Based on the results shown in Fig. 1 and Fig S2, Supporting Information, we proceed to the double pulse orthogonal configuration, which is the main focus of this work.

**Figure 2:**
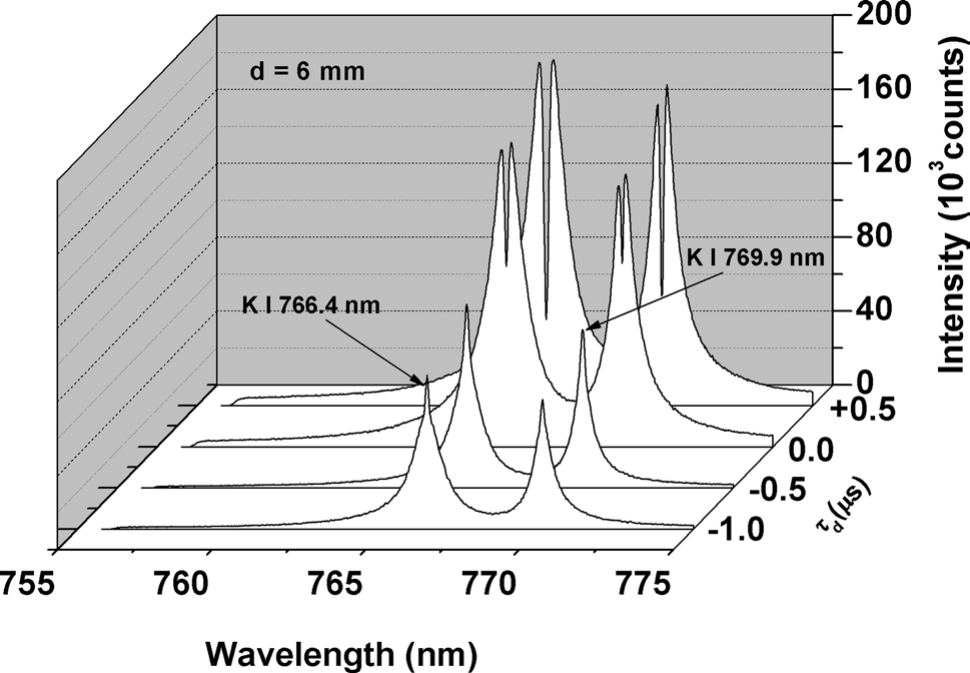
Emission spectra of K I 766.4 nm and K I 769.9 nm from a pure KCl pallet as a function of the inter-pulse delay times (τ_d_) in the double pulse orthogonal configuration at atmospheric air pressure. The distance between the air breakdown plasma and the sample surface (d) is fixed at 6 mm. The first laser energy is fixed at 122 mJ, and the second laser energy is fixed at 20 mJ. The gate delay and gate width of the ICCD are set at 200 ns and 30 µs, respectively, from the second laser initiation.

For the first component of the experiment with a double pulse orthogonal laser, the first laser is operated before and after the initiation of the second laser. The inter-pulse delay time τ_d_ is positive when the second laser is operated prior to the first laser and negative when the first laser is operated prior to the second laser. The gating of the ICCD is determined from the second laser operation and is fixed at 200 ns for gate delay and 30 µs for gate width. The ambient air pressure is the laboratory pressure, which is approximately 101 kPa. The distance between the air breakdown plasma and the sample surface (d) is set at 6 mm. The emission spectra of K I 766.4 nm and K I 769.9 nm from the pure KCl sample as a function of the inter-pulse delay times (τ_d_) are presented in Fig. 2. It is clearly observed that for τ_d_ = 0 µs and 0.5 µs, both emission lines still display a serious self-absorption effect, as indicated by the presence of dip, called self-reversal, at the centre of the lines. When τ_d_ is set at − 0.5 µs and − 1.0 µs, the self-reversal disappears, but the intensity of both emission lines is weakened, and it should also be noted that the FWHM of both lines is still broad compared with the results obtained in Fig. S2(a), Supporting Information. Considering the FWHM of the K I 766.4 nm and K I 769.9 nm emission lines, for the subsequent experiment with d = 6 mm, we use τ_d_ = − 1 µs as the inter-pulse delay time. Note that, in this study, we set the energy of the first laser at 122 mJ because at this value the generated shockwave plasma is strong enough to induce a low air pressure atmosphere near the sample (Fig. S3, Supporting Information). Below this value, the generated shockwave plasma is weak. As a result, the surrounding air pressure is still high, and a self-reverse emission line still present. In fact, in the preliminary experiment, we found that when the first laser energy is set below 122 mJ, for example at 83 mJ, the self-reversal still appears in the emission lines of K and this spectra share the same feature as we obtained when using a single pulse laser (355 nm Nd:YAG laser operating at an energy of 20 mJ) at an air pressure of 10.7 kPa as depicted in Fig. S3, Supporting Information. Meanwhile, above 122 mJ, the shock wave from the first laser will start to ablate the sample. A large amount of material ablation produced by the first and second lasers will generate a thick plasma yielding the appearance of serious self-reversal in the emission lines, as presented in Fig. S3.

**Figure 3:**
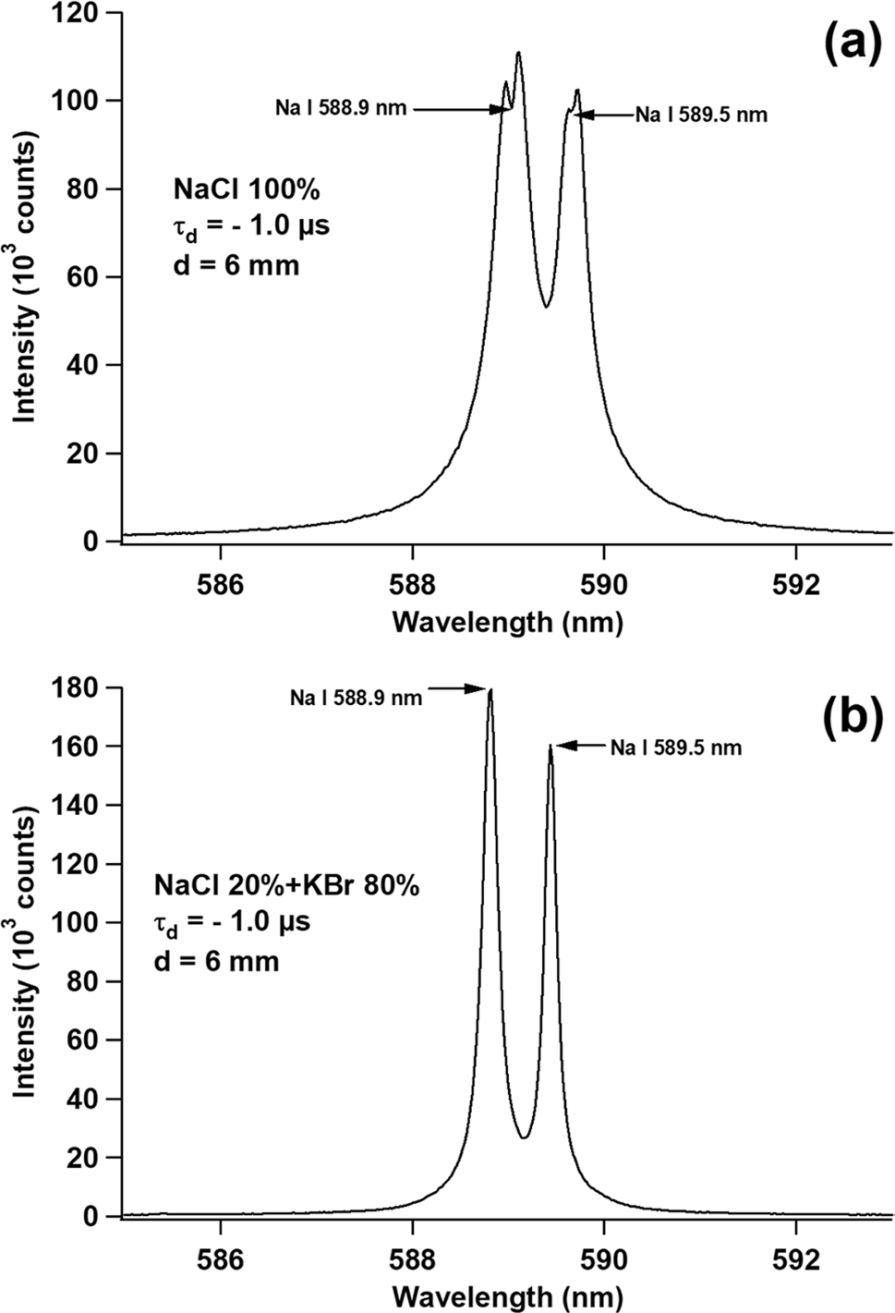
Emission spectra of Na I 588.9 nm and Na I 589.5 nm from (**a**) a pure NaCl pellet and (**b**) a pellet with a mixture of 20% NaCl and 80% KBr at τ_d_ = − 1 µs and d = 6 mm. Other conditions are the same as in Fig. 2.

In the following experiment, we replaced the pure KCl sample with the pure NaCl sample. Figure 3a shows the Na I 588.9 nm and Na I 589.5 nm emission lines obtained by setting τ_d_ to − 1 µs. As shown in Fig. 3a, the self-absorption effect with obvious self-reversal still occurs on both of the emission lines, and the FWHM of both lines is still large. When we reduce the NaCl concentration to 20%, the self-reversal disappears, and sharp and narrow emission lines of Na I were obtained, as shown in Fig. 3b.

**Figure 4:**
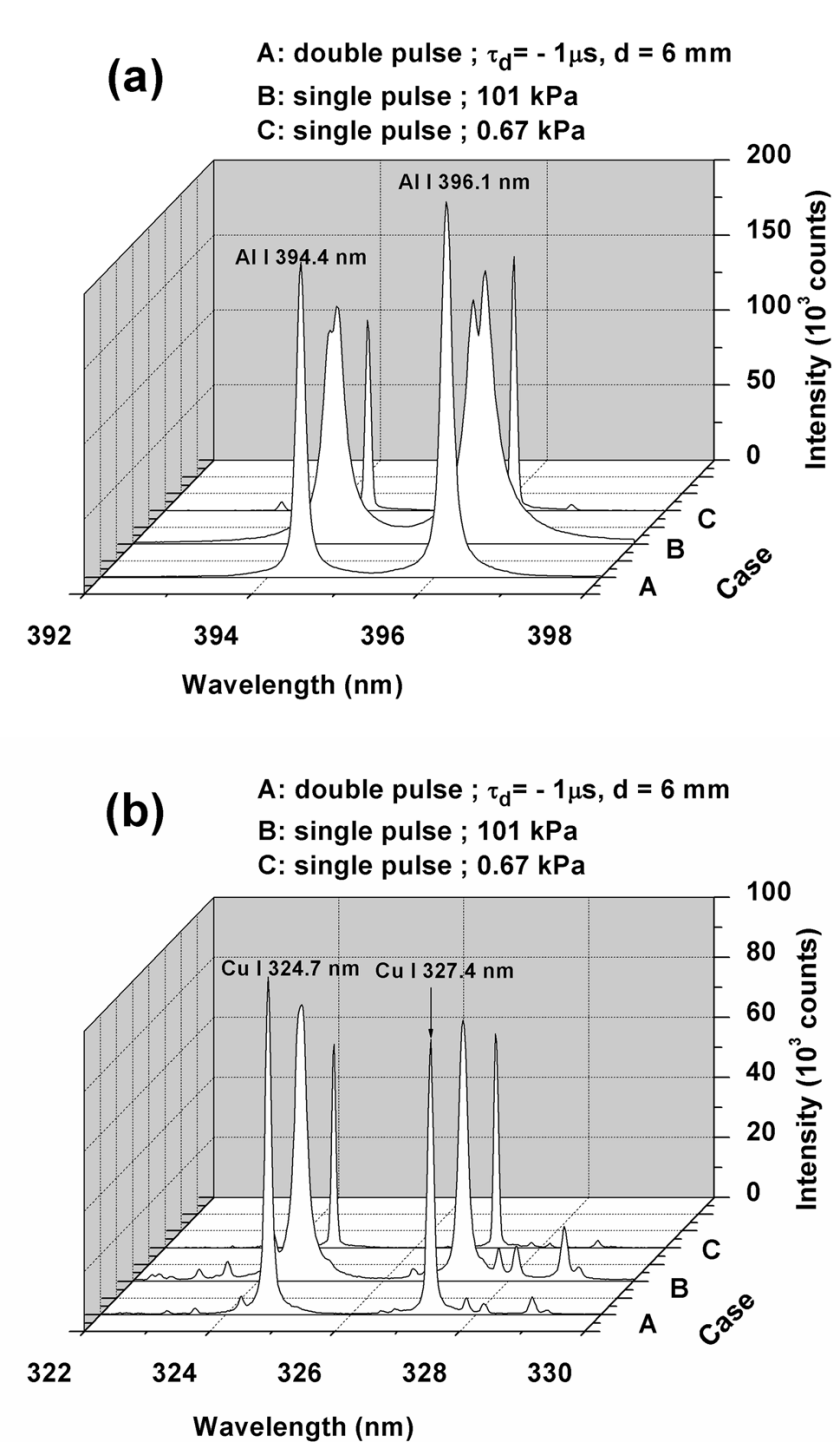
(**a**) Emission spectra of Al I 394.4 nm and Al I 396.1 nm from a pure Al sample under different experimental conditions. A: double pulse with τ_d_ = − 1 µs and d = 6 mm, B: single pulse laser at atmospheric air pressure (101 kPa), and C: single pulse laser at low air pressure (0.67 kPa). For B and C, only the Nd:YAG laser operating at 355 nm with an energy of 20 mJ is used. For A, the first laser energy is fixed at 122 mJ, and the second laser energy is fixed at 20 mJ. The gate delay and gate width of the ICCD are set at 200 ns and 30 µs, respectively, from the second laser initiation. (**b**) Emission spectra of Cu I 324.7 nm and Cu I 327.4 nm from a pure Cu sample under the same conditions as explained in (**a**).

We replaced the NaCl sample with Al and Cu samples to examine whether the above suppression of self-absorption also occurs in metal samples. Following the same best condition for Figs. 2 and 3, the emission lines of Al I 394.4 nm and Al I 396.1 nm are studied. In this case, the result is compared with the result obtained using the single pulse laser at a low air pressure of 0.67 kPa and a high air pressure of 101 kPa, as presented in Fig. 4a. Again, the highest emission intensity of Al I 394.4 nm and Al I 396.1 nm without self-reversal is obtained at the double pulse orthogonal configuration with τ_d_ = − 1 µs. However, we also note that in terms of FWHM, the emission spectra obtained with low pressure plasma are still superior, as indicated by a narrower linewidth. In the case of single pulse laser at atmospheric air pressure, we still find self-absorption with self-reversal in both emission lines.

The emission spectra of Cu I 324.7 nm and Cu I 327.4 nm from the Cu sample are presented in Fig. 4b. No self- reversal is observed for the case of the double pulse orthogonal configuration with τ_d_ = − 1 µs and the single pulse laser at low air pressure. However, again we find that the FWHM of the emission lines obtained from the single laser pulse at low air pressure is still narrower than that of the other two configurations. However, in case of the single pulse laser at atmospheric air pressure, we still observe unclear self-absorption in both emission lines, which is indicated by the blunt emission peak.

**Figure 5:**
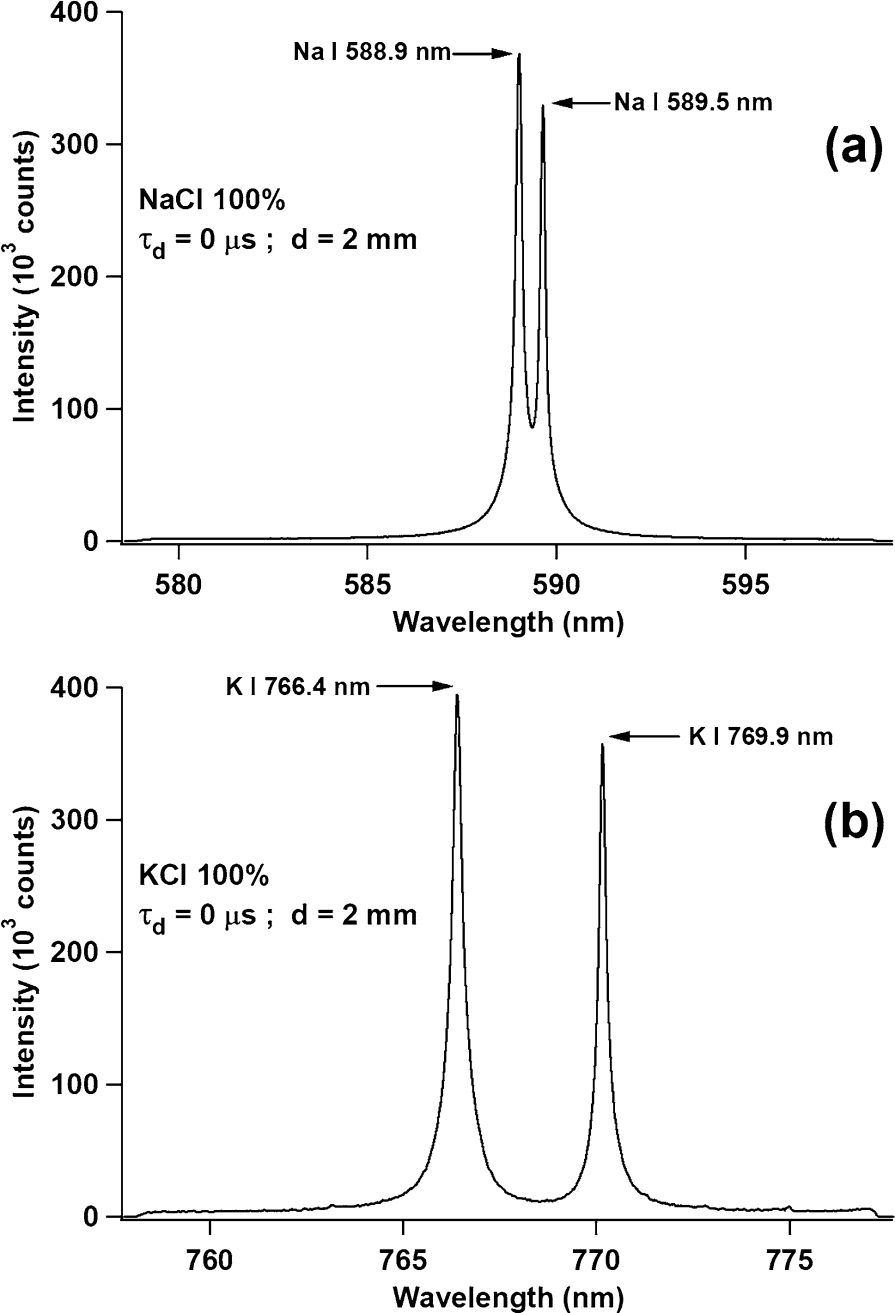
(**a**) Emission spectra of Na I 588.9 nm and Na I 589.5 nm from a pure NaCl pellet at τ_d_ = 0 µs and d = 2 mm. (**b**) Emission spectra of K I 766.4 nm and K I 769.9 nm from a pure KCl pellet at τ_d_ = 0 µs and d = 2 mm. Other conditions are the same as in Fig. 2.

In the subsequent experiment, we changed the distance between the air breakdown plasma and the sample surface (d) from 6 mm (for the experiments in Figs. 1, 2, 3, 4) to 2 mm. Under this condition, with τ_d_ = 0 µs and using the pure NaCl sample, we obtained the emission lines of Na I 588.9 nm and Na I 589.5 nm, as shown in Fig. 5a. As clearly shown in the figure, both emission lines have a strong intensity and narrow FWHM. We also note that this result is much better than the experimental results shown in Fig. 3a or even Fig. 3b. The reason for using τ_d_ = 0 µs is based on the experimental results obtained by varying τ_d_ from − 2 to + 2 µs, where τ_d_ = 0 µs gives the best result. It should be noted that at τ_d_ = 0 µs, the two-laser system is operated simultaneously. This means, at a distance d = 2 mm, we don’t need to use a digital delay generator to synchronize the laser. Again, to test this result, we changed the pure NaCl sample to the pure KCl sample and display the result in Fig. 5b. As can be seen in Fig. 5b, the emission lines of K I 766.4 nm and K I 769.9 nm show a much narrower FWHM and higher emission intensity compared with the previous result shown in Fig. 2. Based on this result, we set d = 2 mm and τ_d_ = 0 µs as the experimental conditions for quantitative analysis.

In Fig. S4, Supporting Information, we summarize the FWHM and emission intensity data for the K, Na, Al, and Cu emission lines obtained from the previous experiment with three experimental conditions, i.e., double pulse orthogonal configuration with τ_d_ = − 1 µs and d = 6 mm in atmospheric air pressure, double pulse orthogonal configuration with τ_d_ = 0 µs and d = 2 mm in atmospheric air pressure, and single laser pulse at 0.67 kPa air pressure. In the case of FWHM, as shown in Fig. S4(a), Supporting Information, the single pulse laser at low air pressure gives the narrowest FWHM for all of the emission lines. The double pulse configuration with d = 2 mm gives a slightly larger FWHM compared with that at d = 6 mm. This is because the presence of air breakdown near the sample surface at d = 2 mm increased ablation amount of the sample. Besides, the presence of air breakdown near the sample surface also induced a strong plasma confinement due to its interaction with the target plasma. These two conditions increased the plasma density and therefore induced more collisional broadening on the emission lines. As a result, the FWHM of the emission lines obtained at d = 2 mm becomes a slightly larger compared with that at d = 6 mm. As shown in Fig. S4(b), Supporting Information, those two conditions also make the emission intensity at d = 2 mm becomes stronger compared with that at d = 6 mm. By combining the data of Fig. S4(a) and Fig. S4(b), Supporting Information, it is natural to choose d = 2 mm for the best condition to suppress self-absorption in this work.

In the next experiment, a series of measurements were conducted on several KCl samples with eight different K concentrations ranging from 0.82 to 52.4 wt%, which were prepared from the mixture of pure KCl with NaCl. The measured K I 766.4 nm emission intensities with respect to the associated K concentrations are plotted in Fig. S5(a), Supporting Information. Each data point in this figure is obtained from the average of 5 data produced by 5 successive laser shots. To evaluate the self-absorption effect on the spectral line, we calculate the self-absorption coefficient (SA) by using the following equation^4,20,21^:

1 $$ SA = \frac{{1 - e^{ - \alpha C} }}{\alpha C} $$

where *C* is the elemental concentration and $\alpha$ is the self-absorption factor that can be deduced from exponential fitting of the spectral intensity as a function of elemental concentration by using the following equation:

2 $$ I(C) = A(1 - e^{ - \alpha C} ) + I_{b} $$

*A* and $I_{b}$ in Eq. (2) represent relative spectral intensity and background intensity, respectively. The value of *A* and $I_{b}$ are also deduced from the exponential fitting parameters of the calibration curve. From the exponential fitting (see Fig. S5(a)), we found that the value of $\alpha$ is 0.0058, indicating negligible self-absorption. The value of exponential fitting $R^{2}$ is 0.998, meaning that the $\alpha$ value obtained using the above calibration model is highly reliable. It is observed that the K concentration and its associated emission intensity exhibit a clearly linear relationship with a very high determination coefficient $R^{2}$ of 0.999 over a wide measurement range of 0.82–26.2 wt% and intercept near zero point. The values of SA of K I 766.4 nm calculated using Eq. (2) with for different K concentrations are shown in Fig. S5(b). The SAs were close to 1 over a wide concentration range of 0.82–26.2 wt%, demonstrate the effectiveness of our proposed method in suppressing the self-absorption effect at atmospheric air pressure.

## Conclusion

In summary, we have shown that the self-absorption effect can be significantly suppressed with the use of a double pulse orthogonal configuration in atmospheric air pressure. We have demonstrated that by proper adjustment of the distance between the air breakdown and sample surface as well as the inter-pulse delay times, the resonance spectrum of a high concentration atom at atmospheric air pressure free from the self- reversal can be realized. In this work, we found that high intensity and self-reversal free resonance lines with very small self-absorption coefficients can be achieved by adjusting the distance between the air breakdown and the sample surface to 2 mm and by firing both lasers at the same time (τ_d_ = 0 µs). We believe that this condition plays an important role in creating a vacuum-like condition near the sample. Using this condition, we succeeded in obtaining a linear calibration curve for K over a wide concentration range of 0.82–26.2 wt% with a nearly zero intercept. This result again proved that our technique is useful and applicable for emission-based spectrochemical analysis when resonance emission lines are chosen as the analytical lines.

## Supplementary information

Supplementary Information.
