# Supplementary material for: Suppression of self-absorption in laser-induced breakdown spectroscopy using a double pulse orthogonal configuration to create vacuum-like conditions in atmospheric air pressure
Source: Sci Rep. 2020 Aug 6;10:13278. doi: 10.1038/s41598-020-70151-6 (PMC7411021; doi:10.1038/s41598-020-70151-6)
Supplement: Supplementary file 1 — Supplementary Information. [file 41598_2020_70151_MOESM1_ESM.pdf]

SUPPORTING INFORMATION

Suppression of self-absorption in laser-induced breakdown spectroscopy using a double pulse orthogonal configuration to create vacuum-like conditions in atmospheric air pressure

Indra Karnadi[1], Marincan Pardede[2], Ivan Tanra[1], Rinda Hedwig[3], Alion Mangasi Marpaung[4], Zener Sukra Lie[5], Eric Jobiliong[2], Dennis Kwaria[6], Maria Margaretha Suliyanti[7], Muliadi Ramli[8], Kurnia Lahna[9], Tjung Jie Lie[6], Hery Suyanto[10], Koo Hendrik Kurniawan[6,*], Kiichiro Kagawa[6,11]

[1]Department of Electrical Engineering, Krida Wacana Christian University, Jakarta 11470, Indonesia.
[2]Department of Electrical Engineering, University of Pelita Harapan, Tangerang 15811, Indonesia.
[3]Computer Engineering Department, Faculty of Engineering, Bina Nusantara University, Jakarta 11480, Indonesia.
[4]Faculty of Mathematics and Natural Sciences, Jakarta State University, Jakarta 13220, Indonesia.
[5]Automotive & Robotics Program, Computer Engineering Department, Binus ASO School of Engineering, Bina Nusantara University, Jakarta 11480, Indonesia.
[6]Research Center of Maju Makmur Mandiri Foundation, Jakarta 11630, Indonesia.
[7]Research Center for Physics, Indonesia Institute of Science, Kompleks Puspiptek, Tangerang Selatan 15314, Indonesia.
[8]Chemistry Department, Faculty of Mathematics and Natural Sciences, Syiah Kuala University, Darussalam, Banda Aceh 23111, Indonesia.
[9]Physics Department, Faculty of Mathematics and Natural Sciences, Syiah Kuala University, Darussalam, Banda Aceh 23111, Indonesia.
[10]Department of Physics, Faculty of Mathematics and Natural Sciences, Udayana University, Kampus Bukit Jimbaran, Denpasar 80361, Indonesia
[11]Fukui Science Education Academy, Takagi Chuo 2 chome, Fukui 910-0804, Japan.

*Author to whom correspondence should be sent.
Email: kurnia18@cbn.net.id

S-1. Experimental Arrangement

Figure S1 shows the experimental arrangement used in this work. In this experiment, the plasma induced by the second laser irradiation has a diameter of around 8 mm. Therefore, we adjust the distance (d) between the air breakdown plasma and the sample surface in the range of 0 to 8 mm.

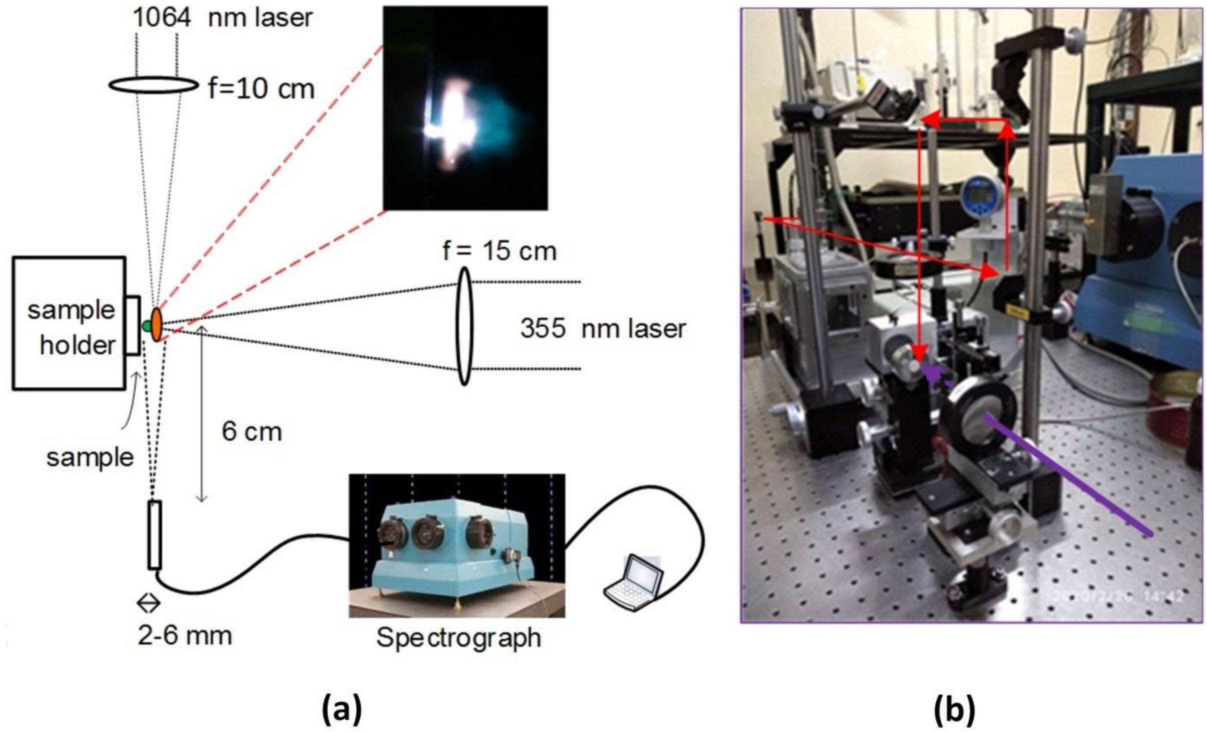

Figure S1. (a) Schematic diagram of the experimental setup used in this work. Inset figures show a photograph of the air breakdown and target plasma. (b) Real apparatus setup. The red line illustrates the light trajectory of the 1,064 nm laser, and the purple line illustrates the light trajectory of the 355 nm laser.

S-2. Emission spectra of K from a pure KCl pellet and Na from a pure NaCl pellet at low air pressure (0.67 kPa)

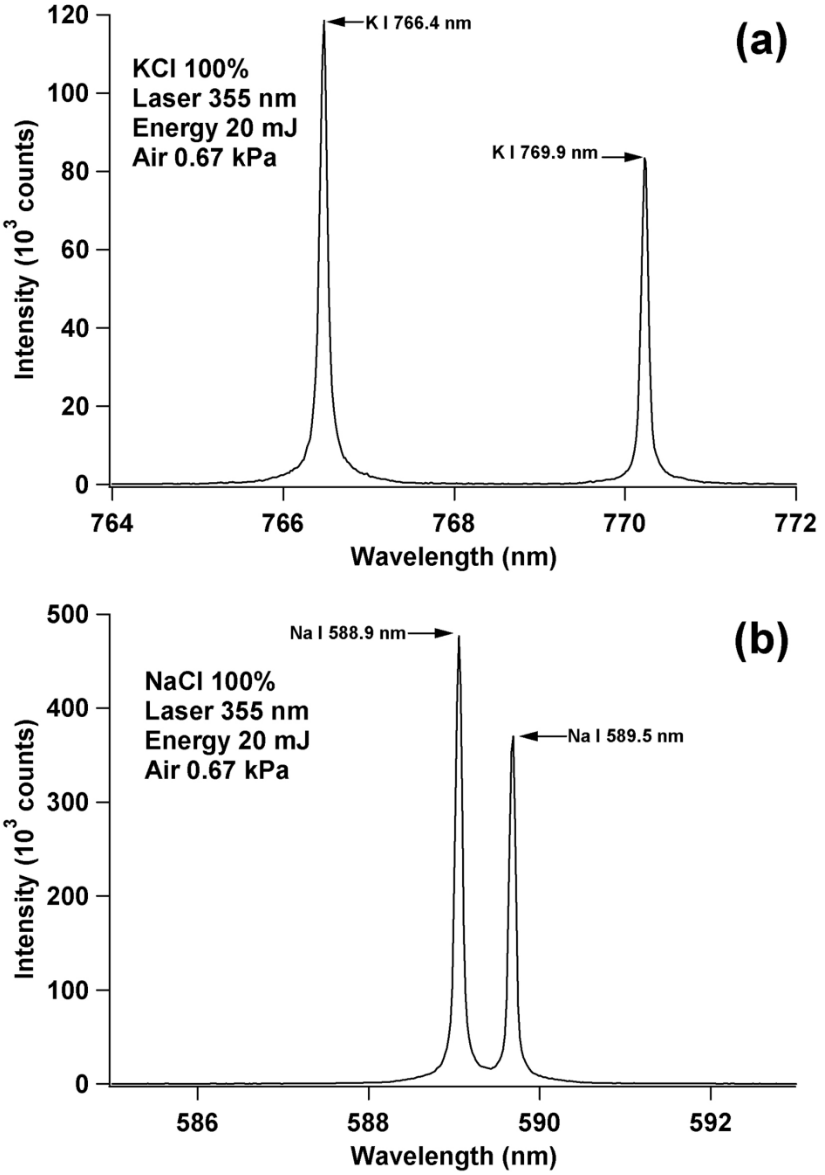

Figure S2. (a) Emission spectra of K I 766.4 nm and K I 769.9 nm from a pure KCl pellet sample and (b) emission spectra of Na I 588.9 nm and Na I 589.5 nm from a pure NaCl pellet sample when irradiated with a Nd:YAG laser at 355 nm wavelength and energy of 20 mJ. The ambient air pressure is 0.67 kPa. The gate delay and gate width of the ICCD are set at 200 ns and 30 µs, respectively.

S-3. Emission spectra of K from a pure KCl pellet obtained using different experimental conditions

The vacuum-like condition inside the laser-induced shockwave can be deduced indirectly by comparing the spectra obtained using the double pulse lasers configuration with the spectra obtained using a single pulse laser at low air pressure. The following figures show the emission spectra of K I 766.4 nm and K I 769.9 nm, obtained from our preliminary experiment by setting the distance between the first laser plasma and the sample surface to 6 mm and the inter-pulse delay to τ_d = -1 μs. Figure S3(a) shows the corresponding emission spectra of K when the energy of the first laser is set at 83 mJ. This spectra share the same feature as we obtained when using a single pulse laser (355 nm Nd:YAG laser operating at an energy of 20 mJ) at an air pressure of 10.7 kPa, as depicted in Fig. S3(b). Based on this result we assumed that the surrounding air pressure is still high. When we increased the energy of the first laser to 122 mJ, we found that the obtained spectra of K (Fig. S3(c)) share the same feature as we obtained when using a single pulse at an air pressure of 2.67 kPa, as shown in Fig. S3(d). And when we further increased the energy of the first laser to 160 mJ, the first laser will start to ablate the sample. A large amount of material ablation produced by the first and second lasers will generate a thick plasma yielding the appearance of serious self-reversal in the emission lines, as presented in Fig. S3(e). Therefore, based on the results obtained from our preliminary study, we choose 122 mJ as the energy for the first laser.

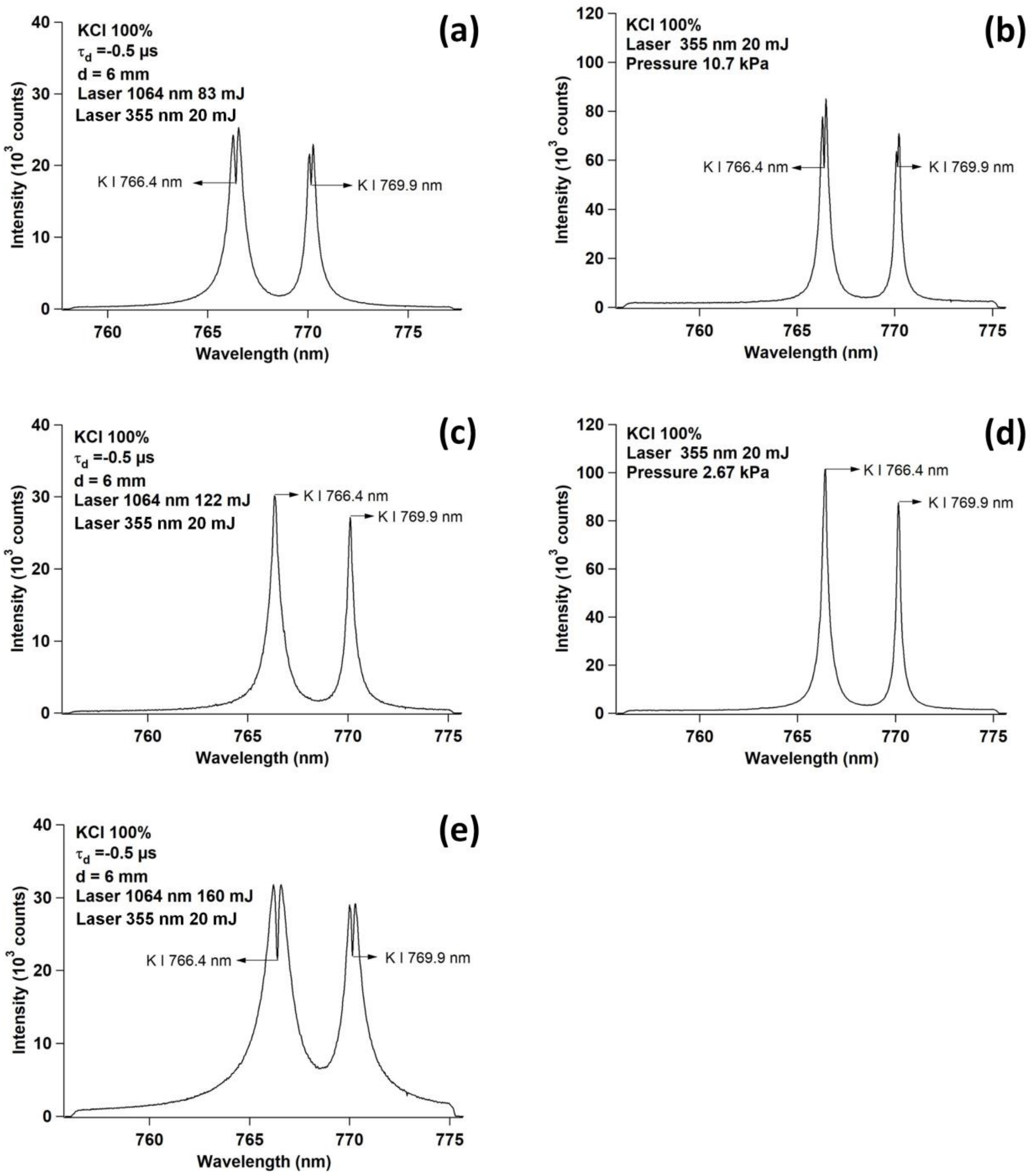

Figure S3. (a), (c), and (e) Emission spectra of K I 766.4 nm and K I 769.9 nm from a pure KCl pellet sample obtained using a double pulse laser configuration with τ_d = - 1 µs and d = 6 mm. The second laser energy is fixed at 20 mJ and the first laser energy is fixed at 83 mJ, 122 mJ, and 160 mJ, respectively. The ambient air pressure is 101 kPa. The gate delay and gate width of the ICCD are set at 200 ns and 30 µs, respectively, from the second laser initiation. (b) and (d) Emission spectra of of K I 766.4 nm and K I 769.9 nm from a pure KCl pellet sample obtained

using a single pulse laser (355 nm Nd:YAG laser operating at an energy of 20 mJ) at an air pressure of 10.7 kPa and 2.67 kPa, respectively. The gate delay and gate width of the ICCD are set at 200 ns and 30 μs, respectively.

S-4. FWHM and emission intensity for different emission lines obtained using different experimental conditions.

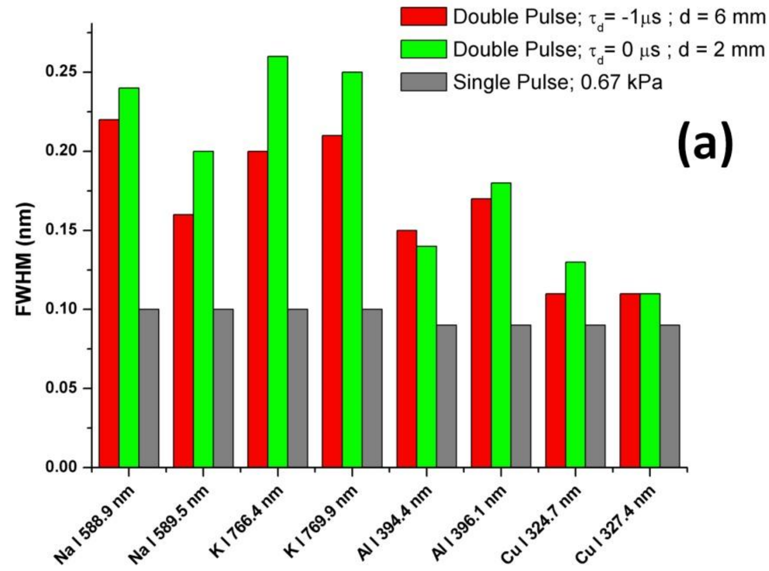

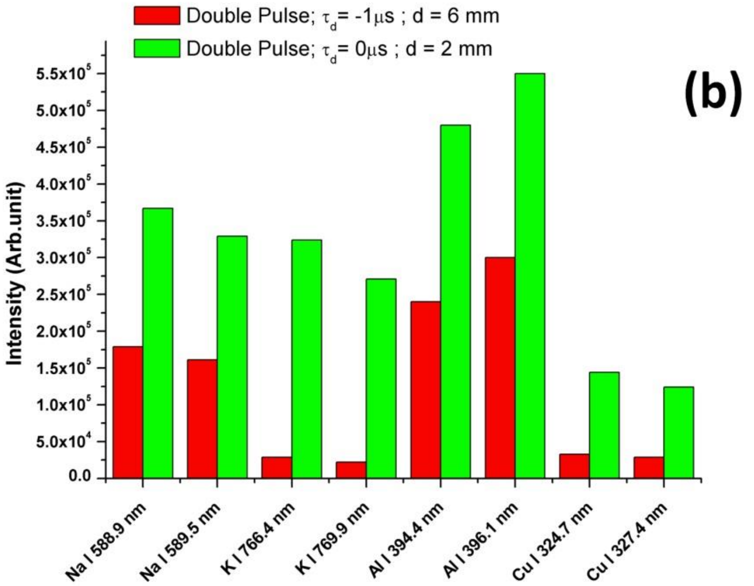

Figure S4. (a) FWHM for different emission lines obtained using a double pulse configuration with τ_d = - 1 μs and d = 6 mm, τ_d = 0 μs and d = 2 mm, and single pulse at low air pressure (0.67 kPa). (b) Emission intensity for different emission lines obtained using a double pulse configuration with τ_d = - 1 μs and d = 6 mm and τ_d = 0 μs and d = 2 mm.

S-5. Calibration curves and self-absorption coefficients of K I 766.4 nm

The measured K I 766.4 nm emission intensities with respect to the associated K concentrations are plotted in Fig. S5(a). To establish the calibration curve, the intensity of the corresponding spectral line is measured by taking the maximum intensity after subtracting the background. The data in the calibration curve can be exponentially fitted using the following equation

$$I(C) = 325504(1 - e^{-0.0058\,C}) + 0.4$$

where C is the elemental concentration. From the exponential fitting, we found that the value of α is 0.0058, indicating negligible self-absorption. The value of exponential fitting R^2 is 0.998, meaning that the α value obtained using the above calibration model is highly reliable. The data in the figure is also fitted with the following linear equation for the concentration range of 0.82-26.2 wt.%.

$$I(C) = 1827\,C + 64$$

It is observed that the K concentration and its associated emission intensity exhibit a clearly linear relationship with a very high determination coefficient R^2 of 0.999 over a wide measurement range of 0.82-26.2 wt.% and intercept near zero point.

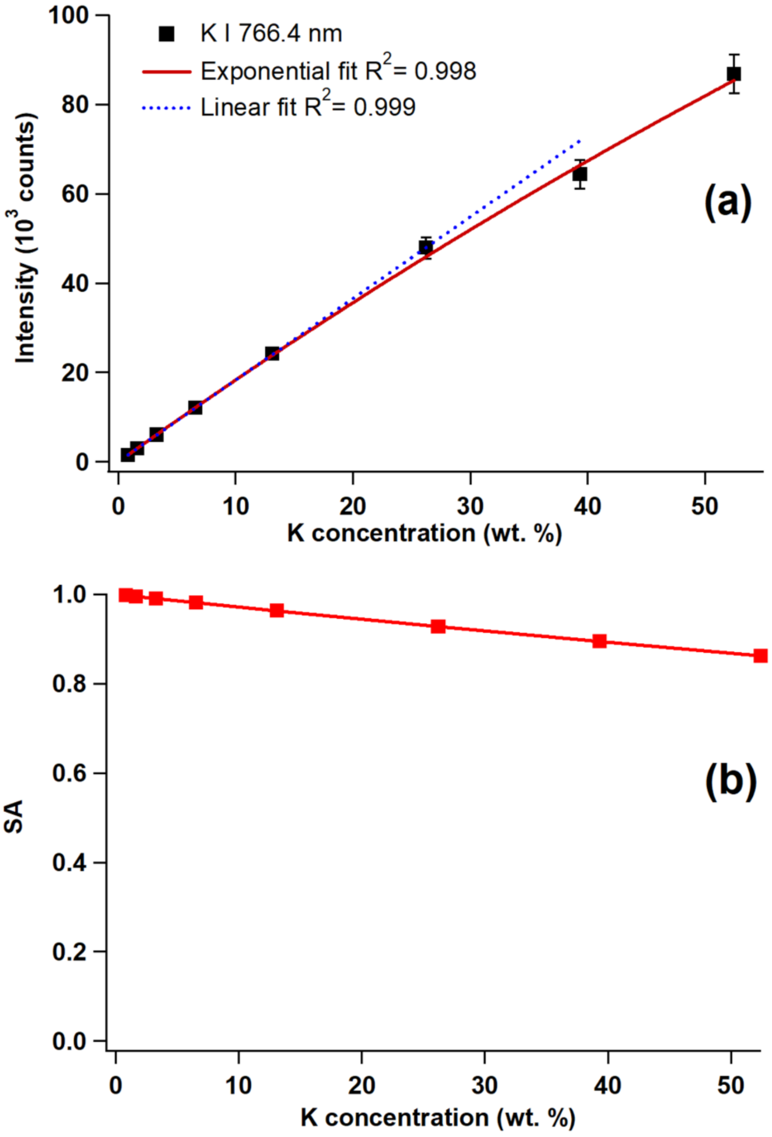

Figure S5. (a) Calibration curve for K I 766.4 nm obtained using double pulse configuration with τ_d = 0 µs and d = 2 mm at atmospheric air pressure. (b) Self-absorption coefficients SAs of K I 766.4 nm in the proposed double pulse orthogonal configuration.
